# Challenges in clinical applications of brain computer interfaces in individuals with spinal cord injury

**DOI:** 10.3389/fneng.2014.00038

**Published:** 2014-09-24

**Authors:** Rüdiger Rupp

**Affiliations:** Experimental Neurorehabilitation, Spinal Cord Injury Center, Heidelberg University HospitalHeidelberg, Germany

**Keywords:** brain computer interface, spinal cord injury, complications, BCI performance, clinical application, neurorehabilitation

## Abstract

Brain computer interfaces (BCIs) are devices that measure brain activities and translate them into control signals used for a variety of applications. Among them are systems for communication, environmental control, neuroprostheses, exoskeletons, or restorative therapies. Over the last years the technology of BCIs has reached a level of matureness allowing them to be used not only in research experiments supervised by scientists, but also in clinical routine with patients with neurological impairments supervised by clinical personnel or caregivers. However, clinicians and patients face many challenges in the application of BCIs. This particularly applies to high spinal cord injured patients, in whom artificial ventilation, autonomic dysfunctions, neuropathic pain, or the inability to achieve a sufficient level of control during a short-term training may limit the successful use of a BCI. Additionally, spasmolytic medication and the acute stress reaction with associated episodes of depression may have a negative influence on the modulation of brain waves and therefore the ability to concentrate over an extended period of time. Although BCIs seem to be a promising assistive technology for individuals with high spinal cord injury systematic investigations are highly needed to obtain realistic estimates of the percentage of users that for any reason may not be able to operate a BCI in a clinical setting.

## INTRODUCTION

In Europe, an estimated number of 330,000 people are living with the consequences of spinal cord injury (SCI), with 11,000 new injuries occurring per year (Ouzký, 2002; van den Berg et al., 2010). Numbers for the United States are in the same range (National Spinal Cord Injury Statistical Center, 2012). Despite marked regional differences across the globe, there has been a trend toward increasing prevalence rates of SCI over the past decades (Furlan et al., 2013). While the most frequent causes of SCI continue to be traffic, work-related and sporting accidents, in industrial countries there is an ongoing trend toward a higher proportion of non-traumatic lesions (Exner, 2004). As a consequence, the average age of persons at the time of injury is steadily increasing (National Spinal Cord Injury Statistical Center, 2012). Depending on its severity the SCI leads to restrictions up to the complete loss of motor, sensory and autonomic functions below the level of injury. Currently, ∼55% of all individuals with an SCI are tetraplegic due to injuries of the cervical spinal cord with resulting life-long paralysis of the lower and upper extremities. The majority of tetraplegic patients (∼28%) have a neurological level of lesion at C4 and C5 at the time of discharge from acute care to rehabilitation facilities (National Spinal Cord Injury Statistical Center, 2012). In lesions at the level of C5, finger function is typically impaired, while in most C4 lesions, hand function and elbow flexion are additionally limited. About 8% of the patients have a neurological level rostral to C4 resulting in the loss of motor functions of both upper extremities including shoulder, elbow, and hand movements. These individuals lose their independence and privacy almost completely, which results in a tremendous decrease in quality of life.

### MEDICAL CONSEQUENCES OF SCI IN THE ACUTE PHASE

A SCI results in impairments of motor, sensory and autonomic functions below the lesion. The degree of initial impairment and the potential for neurological recovery is mainly determined by the severity and location of the lesion.

The first weeks after the injury patients are in the phase of the spinal shock, i.e., that no tendon tap reflexes and flaccid muscle tones are present. The spinal shock typically ends within the first 2 weeks after onset of SCI with reappearing tendon reflexes and muscle tone. After spinal shock spasms, i.e., involuntary muscle contractions that cannot be suppressed or controlled by the patient, as clinical signs of spasticity slowly show up (Hiersemenzel et al., 2000). Spasticity may result in abnormal joint positions and later in joint contractures in particular if motor neurons of antagonistic muscles have been damaged. An example is a fixed elbow joint in fully flexed position after a C4 lesion with a hyperactive biceps and a completely paralyzed triceps muscle.

A variety of autonomic dysfunctions develop after an SCI including paralysis of the bladder and bowel and orthostatic hypotension due to venous pooling of the blood in the paralyzed legs. In individuals with lesions at or above the level of the fourth thoracic spinal segment additional cardiovascular complications such as low systolic and diastolic blood pressure, bradycardia, and autonomic dysreflexia (AD) are present. After spinal shock ends spastic activity may develop in the detrusor muscle restricting the bladder capacity to store urine and resulting in incontinence.

In very high cervical lesions above the level of C3 respiratory problems are present due to impaired voluntary control of the diaphragm. This applies in particular to patients in the acute phase, during which 6.5% of all patients are respirator dependent in the first weeks after injury for at least some hours a day (National Spinal Cord Injury Statistical Center, 2012).

Rehabilitation starts on the first day after the injury. After cervical SCI patients are in need of assistive technology for control of devices such as computers, wheelchairs or environmental control systems. The therapeutic regimes applied in this early phase of rehabilitation mainly focus on restoration of impaired motor functions by inducing spinal and supraspinal neuroplasticity.

### PERSISTENT IMPAIRMENTS IN CHRONIC SCI

The highest degree of neurological recovery occurs within the first 3 months after injury, while functional recovery is delayed to up to 6–12 months (Curt et al., 2008). People with an initial sensorimotor complete [ASIA Impairment Scale A (Waring et al., 2010)] lesion have the lowest potential for substantial neurological and functional recovery, while initially motor incomplete patients have a high probability to regain a relevant ambulatory function. The bilateral loss of the grasp function in individuals suffering from a cervical SCI severely limits the affected individuals’ ability to live independently and retain gainful employment post injury. Therefore, one of the main priorities of these patients is to improve a missing grasping and reaching function (Anderson, 2004; Snoek et al., 2004; Collinger et al., 2013). If there is sufficient voluntary control of muscles distal to the elbow, surgical procedures such as muscle and tendon transfers, tenodesis and arthrodeses, can be successfully applied for regaining a meaningful grasp function (Hentz and Leclercq, 2002; Keith and Peljovich, 2012). However, if no voluntary motor functions distal to the elbow joint are present or an individual is unwilling to undergo surgery with the associated extended post-surgical rehabilitation period, grasp neuroprostheses on the basis of functional electrical stimulation (FES) may represent a valid alternative for restoring upper extremity function (Rupp and Gerner, 2007). If motor impairments persist, they may lead to negative secondary complications that restrict the successful application of grasp neuroprosthesis. Immobility may lead to a reduction in the passive range of motion of affected joints, which may result in severe contractures with totally immobile joints due to calcified joint capsules. Adequate physical therapy may prevent some of these negative side effects on the musculoskeletal body structures. If no voluntary movements are preserved in the upper extremities no restorative approaches are currently available. To compensate for the loss of motor function and to allow individuals with severe disabilities to participate in society, assistive devices are used enabling environmental control and computer, internet, and social media access. The latter is extremely important for end users with severe motor impairments, because in the virtual world persons with handicaps are on the same level than non-impaired people. Examples for assistive devices used for this purpose are – depending on the residual capabilities of the end user – joysticks for the hand or the chin, suck-and-puff control, voice control, or eye-tracking systems. In very high lesioned patients and particularly those depending on artificial ventilation the input devices for setup of an electronic user interface are in general very limited and may not work with a sufficient level of performance over an extended period of time. Therefore, over the last decade BCIs have become an interesting option for end users who achieve only a moderate level of control with traditional input devices.

## BRAIN COMPUTER INTERFACES

Brain computer interfaces (BCIs) are technical systems that provide a direct connection between the human brain and a computer (Wolpaw et al., 2002). These systems are able to detect thought-modulated changes in electrophysiological brain activity and transform the changes into control signals. A BCI system consists of four sequential components: (1) signal acquisition, (2) feature extraction, (3) feature translation, and (4) classification output, which interfaces to an output device. These components are controlled by an operating protocol that defines the onset and timing of operation, the details of signal processing, the nature of the device commands, and the oversight of performance (Shih et al., 2012).

### TECHNOLOGY AND BRAIN SIGNALS OF BCI SYSTEMS FOR CLINICAL APPLICATIONS

Although, all implementations of BCIs build upon the same basic components, they differ substantially in regard to complexity of the technology for acquisition of brain signals, their basic mode of operation (cue-based, synchronous vs. asynchronous) and the underlying physiological mechanisms (Birbaumer et al., 2008; Riccio et al., 2012). For application in the clinical environment non-invasive, small scale systems represent the only realistic option. Most of the non-invasive BCI systems rely on brain signals that are recorded by electrodes on the scalp [electroencephalogram (EEG)]. Another option for practically usable BCIs are systems based on near-infrared spectroscopy (NIRS; Strait and Scheutz, 2014).

Near-infrared spectroscopy uses the fact that the transmission and absorption of near-infrared light in human body tissues contains information about hemoglobin concentration changes. When a specific area of the brain is activated, the localized blood volume in this area changes rapidly. Optical imaging can measure the location and activity of specific regions of the brain by continuously monitoring blood hemoglobin levels through the determination of optical absorption coefficients.

In contrast to NIRS, EEG-based BCI systems can function in most environments with relatively inexpensive equipment and therefore offer the possibility of practical use in either the clinical setting or later in end users’ homes. A variety of EEG signals have been used as measures of brain activity: event-related potentials (ERPs; Farwell and Donchin, 1988; Sellers and Donchin, 2006a; Nijboer et al., 2008), frequency oscillations particularly the EEG sensorimotor rhythms (SMRs; Pfurtscheller and Lopes da Silva, 1999; Wolpaw et al., 2000), slow cortical potentials (SCPs; Birbaumer et al., 1999; Neumann et al., 2003), and steady-state responses (SSRs; Cheng et al., 2002). EEG-based BCIs can be categorized into endogenous, asynchronous and exogenous, synchronous systems. Asynchronous BCIs depend on the users’ ability to voluntary modulate their electrophysiological activity such as the EEG amplitude in a specific frequency band. In asynchronous BCIs the time point for changes of the control signals is not predefined by the system, but the user is free to initiate decisions at any time. These systems usually require a substantial amount of training. Examples for this class of BCIs are systems based on the detection of SMRs or SCPs. Synchronous BCIs depend on the electrophysiological activity evoked by external stimuli and do not require intensive training. The most common synchronous BCI is based on P300 ERPs. Although systems based on steady-state evoked potentials (SSEPs) such as steady-state visual evoked potentials (SSVEPs) or steady-state somatosensory evoked potentials (SSSEPs) combine components of asynchronous and synchronous approaches, the introduction of cues improves their accuracy. Depending on the brain signals used for operation BCIs greatly vary in regard to the minimal and typically used number of electrodes, training times, accuracies, and typical information transfer rates (for overview see **Table 1**; Birbaumer et al., 2003; Hinterberger et al., 2004; Guger et al., 2012a; Combaz et al., 2013).

**Table 1:** Types of EEG-based BCIs suitable for application in patients in the acute phase after SCI together with their main characteristics.

Parameter BCI	Minimal (typical) number of electrodes	Training time	Population with 90–100 (below 80) accuracy without training (%)	Typical rate of decisions/min
SMR (2-class)	4 (10) + 1 reference	weeks to months	6 (81)	4 Bits/min
SCP	1 (1) + 2 reference	weeks to months	33 with accuracy above 70	<1 Bit/min
P300	3 (9) + 1 reference	minutes to <1 h	73 (11)	10 Bits/min
SSVEP	6 + 1 reference	minutes to <1 h	87 (4)	12 Bits/min

*An overview of the most common practical types of BCIs together with their minimal number of electrodes, a qualitative estimation of typical training times and their typical accuracy and bit rate is provided. A common ground electrode, which is needed for all BCIs, is not explicitly mentioned.*

#### BCIs based on slow cortical potentials

Slow cortical potentials are slow voltage changes generated on the cerebral cortex, with a duration varying between 300 ms and several seconds. Negative SCPs are typically associated with movement and other functions that imply cortical activity. It has been demonstrated that people are able to self-regulate these potentials and use these modulations for control of assistive devices like a spelling device (Rockstroh et al., 1984). By this, an alternative communication channel was provided to totally paralyzed patients. However, with SCP-based BCIs only a very low information transfer rate of typically less than one letter per 2 min can be achieved (Birbaumer et al., 1999). Additionally, a substantial amount of training, during which patients receive feedback about their EEG-activity, is necessary to achieve a sufficient level of control. Therefore, SCP-based BCIs do not represent the first choice for providing individuals with high SCI with a communication or control interface in the acute phase after the injury.

#### BCIs based on sensorimotor rhythms

Another type of EEG-based BCI exploits the modulation of SMRs. These rhythms are oscillations in the EEG occurring in the alpha (8–12 Hz) and beta (18–26 Hz) bands and can be recorded over the primary sensorimotor areas on the scalp. Their amplitude typically decreases during actual movement and similarly during mental rehearsal of movements [motor imagery (MI); Pfurtscheller and Lopes da Silva, 1999; Neuper et al., 2005]. Several studies have shown that people can learn to modulate the SMR amplitude by practicing MIs of simple movements, e.g., hand/foot movements (Kaiser et al., 2014; Toppi et al., 2014). This process occurs in a closed-loop, meaning that the system recognizes the SMR amplitude changes evoked by MI and these changes are instantaneously fed back to the users. This neurofeedback procedure and mutual human–machine adaptation enables BCI users to control their SMR activity and use these modulations to control output devices in an asynchronous manner (Pineda et al., 2003; Cincotti et al., 2008).

For a typical 2-class SMR-BCI different paradigms of MIs such as one hand vs. feet or left vs. right hand are used either in a switch based fashion by introduction of a threshold or in an analog manner by directly connecting the classifier output to the output device. An often underestimated problem in practical applications of BCIs and in particular of SMR-based BCIs is the detection of a non-intention condition, during which a user does not want to send any command (zero-class). This so called zero-class problem is often handled in brain-switch implementations by defining one MI class as the resting class or to use long MIs to pause or reactivate the system (Pfurtscheller et al., 2003; Rohm et al., 2013). However, this approach is not appropriate for all applications, which renders the zero-class problem as one of the major limiting factors for practical use of BCIs.

Motor imagery-brain computer interfaces offer further possibilities in the context of neurorehabilitation of spinal cord injured patients that go beyond the traditional use for control of assistive device. After a SCI substantial functional brain reorganization occurs that plays a critical role for functional recovery and may have pathological consequences (Nardone et al., 2013). The basis for a therapeutic use of BCIs is formed by the fact that the central nervous system shows a life-long ability for neural plasticity, which can be enhanced after a trauma or injury by task-specific training (Dietz and Fouad, 2014). The key elements for an effective neurorehabilitative training based on motor learning are voluntarily triggered movement intentions and a synchronized sensory and proprioceptive feedback of the limbs’ motor actions. BCIs hold promise to enable the detection of intended movements, e.g., the hand, even in high spinal cord injured patients, making them an ideal tool for closed-loop neurorehabilitative therapies when used in combination with grasping and reaching neuroprosthesis (Jackson and Zimmermann, 2012; Rupp et al., 2013; Savic et al., 2014). Additionally, by practicing feedback-controlled MI of paralyzed limbs the integrity of cortical neuronal connections may be preserved or neurological recovery of motor function may be even enhanced (Kaiser et al., 2014).

#### BCIs based on event-related potentials

Event-related potential-based BCIs make use of the fact that specific neural activity is triggered by and involved in the processing of specific events. These systems are implemented with an oddball paradigm, wherein a rare target (oddball event) is presented within frequent non-target events. These BCIs usually exploit an endogenous ERP component, known as P300, as input signal. The P300 is a positive deflection in the EEG occurring 200–500 ms after the presentation of the rare visual, auditory or somatosensory stimulus and is a reliable, easy to detect ERP (Sutton et al., 1965). By focusing attention on the rare target, e.g., by keeping a mental count of its occurrence, the P300 amplitude can be increased and therefore its detection and classification improves (Kleih et al., 2011). In individuals with SCI eye-gaze is preserved and thus a visual rather than an auditory oddball paradigm is the preferred choice, because the information transfer rate and accuracy is substantially higher and perceived workload much lower in visual P300-based BCIs (Furdea et al., 2009; Halder et al., 2010; Kathner et al., 2013). The big advantage of P300 compared to SMR-based BCIs is that they can be operated with almost no setup time in 99% of the general population (Guger et al., 2009b). Although, P300-BCIs basically work without electrodes on the occipital cortex, their performance can be improved, if electrodes on the posterior head region are used (Krusienski et al., 2008). Special care must be taken that these electrodes do not cause any discomfort in acute patients with high SCI lying in bed and resting their heads on a pillow or using a head-rest.

#### BCIs based on steady-state evoked potentials

Steady-state evoked potentials are stable oscillations that can be elicited by rapid repetitive (usually > 6 Hz) visual, auditory, and somatosensory stimuli. The most common type of SSEP-based BCI are the SSVEP-based BCIs, where screen objects flickering at different frequencies are visually presented to subjects. Focusing their attention to the intended stimulus elicits enhanced SSVEP responses at the corresponding frequency, which can be detected, classified and translated into control commands (Vialatte et al., 2010). SSVEP-based BCIs have the advantages of a high information transfer rate, practically no training time, and they work in almost every user (Allison et al., 2010; Guger et al., 2012a). SSVEPs are recorded over occipital brain areas and the same caution has to be taken like in some P300-based systems to avoid any discomfort caused by electrodes on the back of the head.

A relatively new approach in BCI is the use of auditory steady-state responses (ASSR), where the user can modulate the ASSR by selective attention to a specific sound source such as tone burst trains with different beat frequencies on the left and right ear (Kim et al., 2011). The frequency of the tone, on which a user is putting attention to, can be registered in the EEG and further used to generate a switch signal. Nevertheless, BCIs based on visual evoked potentials are the preferred choice in individuals with SCI that have unimpaired visual function, because the information transfer rate of ASSR-based BCIs is tenfold lower than of SSVEP-based systems (Baek et al., 2013).

The limitations of the placement of electrodes in the posterior region of the skull may be overcome in BCIs based on SSSEPs (Muller-Putz et al., 2006), which record EEG activity over the sensorimotor cortex of the midbrain. In SSSEP-based BCIs tactile stimulators on both hands are used to induce “resonance”-like frequencies in the somatosensory cortex. Users can be trained to modulate these SSSEPs, thereby generating binary control signals. Although they represent an exciting alternative to traditional BCI approaches, SSSEP-based BCIs are in general not applicable in patients with high SCI due to the impairment of sensory functions present in all limbs.

### HYBRID BCIs

A novel development in BCI research is the introduction of the hybrid BCI (hBCI) concept (Müller-Putz et al., 2011). A hBCI consists of a combination of several BCIs or a BCI with other input devices (Allison et al., 2012). These input devices may be based on the registration of biosignals other than brain signals, such as electromyographic activities. Using this approach, a user can generate a single command signal either by fusing different input signals or by simply selecting one of them (Müller-Putz et al., 2011). In the latter case, the input signals can be dynamically routed based on their reliability, i.e., continuously monitoring the quality, and the input channel with the most stable signal will then be selected (Kreilinger et al., 2011). In the case of signal fusion, each of the input signals contributes to the overall command signal with a dedicated weighting factor (Leeb et al., 2011). These factors are generally not static, but can be dynamically adjusted in accordance with their reliability, which is quantified by appropriate quality measures. The hBCI is fully compliant with the user-centered design concept (ISO, 2010). The key message of this approach is that the technology has to be adapted to the individual users’ ability and needs and not vice versa. Combining BCIs with established user interfaces may allow more end users to control assistive technology or may simplify the use of existing devices. However, this extension of the target population comes with the drawback that longer preparation times are needed for setup of the additional components of the hBCI. From the users’ perspective it is important to carefully evaluate the design of the hBCI’s control scheme and not to cause additional mental workload. Control schemes based on a sequential control task of the different input signals are – at least at the beginning of the training – superior to those, for which a user must control different input signals simultaneously. With practice users might learn to perform multiple tasks, thereby making full use of the hBCI approach.

In any case, the hBCI concept helps to overcome limitations inherent to a singular BCI system, e.g., false-positive, unintended decisions or the zero-class problem. In fact the second input signal can be effectively used to indicate an “idling” state or to introduce a context-specific correction mechanism. An example for demonstration of the superiority of this approach is an hBCI-controlled telepresence robot, where the user navigates to the left and right by imagination of movements of the left and right hand and stops/starts the movements of the robot by an electromyographic switch activated by a short muscle twitch (Carlson et al., 2013). In an hBCI controlled communication application based on two BCIs (P300 and SSVEP) SSVEP activity is used to assess whether the subject is focused on a spelling task. If no SSVEP activity is found, then the system assumes that the user is not paying attention to the spelling system and does not output any characters (Panicker et al., 2011). Another example is an hBCI-controlled reaching and grasping neuroprosthesis, in which the hBCI consists of an SMR-BCI combined with an analog shoulder joystick (Rohm et al., 2013). The neuroprosthesis is activated/deactivated by a long MI detected by an SMR-BCI and the degree of hand closing/elbow flexion is controlled by shoulder movements. To prevent an unintended deactivation of the system several context-specific plausibility checks were implemented in the control concept, e.g., deactivation is not allowed, if the hand is closed or if the shoulder is moved. In another example of an hBCI-controlled computer interface based on an SMR-BCI and a mouth mouse, a brain-switch simulating a double-click can only be generated while the mouse cursor is not moving (Faller et al., 2012). This comprehensive list of examples shows that the hBCI concept is a valuable extension of traditional BCI approaches and represents a big step forward toward the regular use of BCIs as assistive devices.

### APPLICATION OF BCIs IN END USERS WITH MOTOR IMPAIRMENTS

Most of the results in BCI research have been obtained involving healthy subjects, in particular students working in research labs due to their easy availability and intrinsic motivation to participate in experiments designed and set up by their own (Moghimi et al., 2013). Only a low percentage (estimated <5%) of BCI studies involved end users with a real need for a BCI, most of them end users with amyotrophic lateral sclerosis (ALS) in the so-called locked-in-state with no motor functions preserved except eye movements (Pasqualotto et al., 2012). All BCI research in end users with SCI was carried out so far with individuals in the chronic stage. This means, that they were participating in studies at the earliest 1 year after the onset of the injury in a stable neurological, psychological, and social state.

#### BCIs for communication

Nowadays, researchers mostly work with the P300 signal for communication purposes. Numerous clinical studies confirm the efficacy of the P300-BCI in paralyzed patients with four choice responses, e.g., “Yes/No/Pass/End” (Sellers and Donchin, 2006b) or “Up/Down/Left/Right” for cursor movement (Piccione et al., 2006; Silvoni et al., 2009). With P300-spellers words could be composed letter by letter, which are arranged in a matrix fashion in rows and columns. One letter is selected by implementation of an oddball paradigm, in which rows and columns are highlighted randomly while the user focuses on one specific letter (target letter) she or he wishes to spell and tries to ignore all other letters that are highlighted in other rows or columns (non-target letters). Each time the target letter is highlighted, a P300 signal occurs in the frontoparietal brain region. Each target letter can be identified by a classifier, which detects the occurrence a P300 signal every time the row and column of the intended letter is highlighted and selects the letter accordingly. In a recent study a new paradigm was recently introduced for enhancement of the P300 control (Kaufmann et al., 2013), in which a famous face – in this case the face of Albert Einstein is superimposed – on top of the matrix display. By the implementation of this paradigm persons formerly unable to control a traditional P300-based speller were enabled to successfully use this kind of communication interface.

An alternative to P300 based spellers are SMR-based spelling systems such as the Hex-o-Spell paradigm (Blankertz et al., 2006). In the Hex-o-Spell paradigm hexagons filled with groups of letters or a single letter are arranged in a circular fashion with a pointing arrow in the center of the circle. The circle can be rotated by one type of MI, e.g., right hand movements, and extended for selection with another MI, e.g., foot movements.

Although, the traditional matrix-based P300-based spellers are the most widespread type of BCIs used for communication purposes, alternative BCIs using different designs and signal modalities such as SSVEPs are developed to build a faster, more accurate, less mentally demanding, and more satisfying BCI (Combaz et al., 2013). Such systems are not only beneficial in end users in a locked-in state, but may also enable basic communication in individuals with very high SCI, who are ventilator dependent. However, this needs to be proven in future clinical studies.

#### BCIs for wheelchair and environmental control

Being mobile is beside communication and manipulation an essential need of motor impaired end users. Wheelchairs represent a very important assistive device to enable mobility in individuals with SCI. Persons with severe motor disabilities are dependant on electrical wheelchairs controlled by hand- or chin-operated manual joysticks. If not enough residual movements are present, eye-gaze or suck-and-puff control units may serve as a wheelchair user interface. Suck-and-puff control is mainly based on four types of commands. If air is blown into/sucks from the device with high pressure/vacuum, the controller interprets this as a forward/backward drive signal. If a low pressure or vacuum is applied, the wheelchair drives right or left. With this rather simple control scheme users are able to perform most navigation tasks with their wheelchair. Though the thresholds for low/high pressure are individually calibrated, the end user must be able to reliably generate two different levels of air pressure/vacuum over a sustained period of time to achieve a good level of control. Since these prerequisites are not present in all very high lesioned spinal cord injured people, BCIs may represent an alternative control option.

At the current state of the art all types of non-invasive BCIs are providing only a limited command rate and are insufficient for dexterous control of complex applications. Thus, before the successful application of control interfaces with low command rates – including BCIs – in mobility devices intelligent control schemes have to be implemented. Ideally, the user only has to issue basic navigation commands such as left, right and forward, which are interpreted by the wheelchair controller integrating contextual information obtained from environmental sensors. Based on these interpretations the wheelchair would perform intelligent maneuvers including obstacle avoidance and guided turnings. In conclusion, in such a control scheme the responsibilities are shared between the user, who gives high-level commands, and the system, which executes low-level interactions with more or less degree of autonomy. With this so called shared control principle researchers have demonstrated the feasibility of mentally controlling complex mobility devices by non-invasive BCIs, despite its slow information transfer rate (Flemisch et al., 2003; Vanhooydonck et al., 2003; Carlson and Demiris, 2008).

Although asynchronous, spontaneous BCIs like SMR-based BCIs seem to be the most natural control option for wheelchairs, there are a few applications using synchronous BCIs (Iturrate et al., 2009; Rebsamen et al., 2010). Like in most communication applications these BCIs are based on the detection of the P300 potential evoked by concentrating on a flashing symbol in a matrix. For wheelchair control the system flashes a choice of predefined target destinations several times in a random order and finally the stimulus that elicits the largest P300 is selected as the target. Afterward the intelligent wheelchair drives to the selected target autonomously. Once there it stops and the subject can select another destination. The fact that the selection of a target takes ∼10 s and that the user intent is only determined at predefined time points takes the usability of cue-based BCIs for control of mobility devices into question.

In BCI-controlled mobility devices developed in the framework of recent European projects MAIA and TOBI the users’ mental intent was estimated asynchronously and the control system provided appropriate assistance for wheelchair navigation. With this approach the driving performance of the BCI controlled device greatly improved in terms of continuous human–machine interaction and enhanced practicability (Vanacker et al., 2007; Galán et al., 2008; Millán et al., 2009; Tonin et al., 2010). In the most recent approach of shared control the user asynchronously sends – with the help of a MI based BCI – high-level commands for turning left or right to reach the desired destination. Short-term low-level interaction for obstacle avoidance is done by the mobility device autonomously. In the applied shared control paradigm the wheelchair pro-actively slows down and turns for avoidance of obstacles as it approaches them. For provision of the latter functionality the wheelchair is equipped with proximity sensors and two webcams for obstacle detection (Borenstein and Koren, 1991; Carlson and Millán, 2013). Cheap webcams were used instead of an expensive laser range-finder to provide an affordable solution, in which the additional equipment for implementation of the shared control does not cost more than the wheelchair itself.

Although a lot of literature is available on the technical specifications of BCI-controlled wheelchairs, only a few studies involving end users are available (Nguyen et al., 2013) and even less involving end users in real need of a BCI.

In the early phase of rehabilitation patients with a cervical spinal injury may not be cardiovascular stable. Therefore, wheelchair mobilization may be difficult and other ways to provide some form of independence and social inclusion need to be found. Access to computers in general and to the internet and social media in particular is an important goal for patients to communicate with their relatives and friends. For this purpose, P300-based BCIs may offer a quick way to setup an interface for assessing traditional social media like Twitter or moving avatars in virtual reality environments like Second Life (Fazel-Rezai et al., 2012). However, the preliminary results obtained in experiments with non-motor impaired persons need to be confirmed in paralyzed end users.

Another important issue is to allow severely paralyzed patients to control their environment independently, to which BCIs-controlled environment control systems may contribute significantly. First results in end users with handicaps show that environmental control by an asynchronous P300 BCI is possible. However, system testing also revealed that the minimum number of stimulation sequences needed for correct classification had a higher intra-subject variability in end users with respect to what was previously observed in young, non-disabled controls (Aloise et al., 2011). Also special focus must be put on the design of the visual control interface to achieve high accuracy while keeping mental effort low (Carabalona et al., 2012). A major progress can be expected in respect to the availability of enhanced BCI-controlled computer and social media access and environmental control from the European projects BrainAble and BackHome.

#### BCIs for control of upper extremity neuroprosthesis

Today, the only possibility of restoring permanently restricted or lost functions to a certain extend in case of missing surgical options (Hentz and Leclercq, 2002) is the application of FES. Over the last 20 years FES systems with different level of complexity were developed and some of them introduced into the clinical environment (Popovic et al., 2002). These systems deliver short current impulses eliciting physiological action potentials on the efferent nerves, which cause contractions of the innervated, yet paralyzed muscles of the hand and the forearm (van den Honert and Mortimer, 1979). On this basis FES artificially compensates for the loss of voluntary muscle control.

When using the FES in a compensatory setup at a very early stage of primary rehabilitation the easiest way of improving a weak or lost grasp function is the application of multiple surface electrodes. With only seven surface electrodes placed on the forearm two grasp patterns, namely lateral grasp and palmar grasp, can be restored (Rupp et al., 2012). With the combination of surface electrodes and a finger synchronizing orthosis the difficulties with daily reproduction of movements and huge variations of grasp patterns depending on wrist rotation angle may be overcome (Leeb et al., 2010).

Through the last decade it has become obvious that the user interface of all current FES devices is not optimal in the sense of natural control, relying on either the movement or the underlying muscle activation from a non-paralyzed body part to control the coordinated electrical stimulation of muscles in the paralyzed limb (Kilgore et al., 2008; Moss et al., 2011). In the case of individuals with a high, complete SCI and the associated severe disabilities not enough residual functions are preserved for control. This has been a major limitation in the development of a reaching neuroprostheses for individuals with a loss not only of hand and finger but also of elbow and shoulder function.

Several BCI approaches mainly based on SSVEPs have been introduced as a substitute for traditional control interfaces for control of an abdominal FES system (Gollee et al., 2010), a wrist and hand orthosis (Ortner et al., 2011) or a hand and elbow prosthesis (Horki et al., 2010).

Apart from those simple approaches, BCIs have enormous implications providing natural control of a grasping and reaching neuroprosthesis control in particular in individuals with a high SCI by relying on volitional signals recorded from the brain directly involved in upper extremity movements.

In Pfurtscheller et al. (2003) a pioneering work showed for the first time that a MI-BCI control of a neuroprosthesis based on surface electrodes is feasible. In this single case study the restoration of a lateral grasp was achieved in a tetraplegic subject, who suffers from a chronic SCI with completely missing hand and finger function. The end user was able to move through a predefined sequence of grasp phases by imagination of foot movements detected by a brain-switch with 100% accuracy. He reached this performance level already prior to the experiment by some months of training with the MI-BCI (Pfurtscheller et al., 2003) and has maintained it for almost a decade by regular continuation of the training (Enzinger et al., 2008).

A second feasibility experiment has been performed, in which a short-term BCI-training has been applied in another individual with tetraplegia. This subject was using a Freehand system for several years. After 3 days of training the end user was able to control the grasp sequence of the implanted neuroprosthesis with a moderate, but sufficient performance (Müller-Putz et al., 2005).

In these first attempts the BCI was rather used as a substitute for the traditional neuroprosthesis control interface than as an extension. With the introduction of FES-hybrid orthoses it becomes more important to increase the number of independent control signals. With the recent implementation of the hBCI framework it became feasible to use a combination of input signals rather than BCI alone. In a first single case study a combination of a MI-BCI and an analog shoulder position sensor is proposed (Rohm et al., 2013). By upward/downward movements of the shoulder the user can control the degree of elbow flexion/extension or of hand opening/closing. The routing of the analog signal from the shoulder position sensor to the control of the elbow or the hand and the access to a pause state is determined by a digital signal provided by the MI-BCI. With a short imagination of a hand movement the user switches from hand to elbow control or vice versa. A longer activation leads to a pause state with stimulation turned off or reactivates the system from the pause state. With this setup a highly paralyzed end user, who had no preserved voluntary elbow, hand and finger movements, was able to perform several activities of daily living, among them eating a pretzel-stick, signing a document and eating an ice cone, which he was not able to perform without the neuroprosthesis.

### CLINICAL APPLICATIONS OF BCIs

In the clinical setting the main focuses of BCIs in patients with an acute or subacute SCI in the first months after injury are (1) the compensation of a temporarily or permanently impaired motor function, preferably if simpler techniques do not allow for a sufficient control of assistive devices, and (2) the maintenance of cortical connectivity for avoidance of maladaptive plasticity with symptoms like neuropathic pain and enhancement of functional recovery by induction of beneficial neuroplasticity (Grosse-Wentrup et al., 2011). Almost all patients with substantial motor impairments are potential candidates for neurofeedback, i.e., receiving feedback on neural cortical states, and neurorehabilitative therapies, e.g., BCI-controlled FES (Birbaumer et al., 2009). Unfortunately, the empirical evidence for a positive impact of BCI technology for therapeutic purposes is scarce and clinical studies are urgently needed to provide evidence for their added value.

For compensation of motor impairments the preferred target population is the group of high lesioned, tetraplegic patients with severe motor impairments in particular of the upper extremities, who may be temporarily ventilator dependent and have limited ability to speak due to the use of a tracheal tube. Most of the BCI research related to communication and control in end users with disabilities has been carried out with individuals in the chronic stage meaning that most of the people returned to their homes, were in a stable neurological and psychological condition and their family members or caregivers were properly instructed to correctly setup and operate a BCI. In contrast to this the condition of the patients and the environment is very different in the clinical setting, which presumably affect the users’ (end users and caregiver) priorities (Huggins et al., 2011).

The aim of the following chapter is to provide an overview of factors that may limit the successful implementation of BCIs for control of assistive devices or for neurorehabilitation in the clinical setting.

## FACTORS LIMITING THE CLINICAL APPLICATION OF BCIs

**FIGURE 1:**
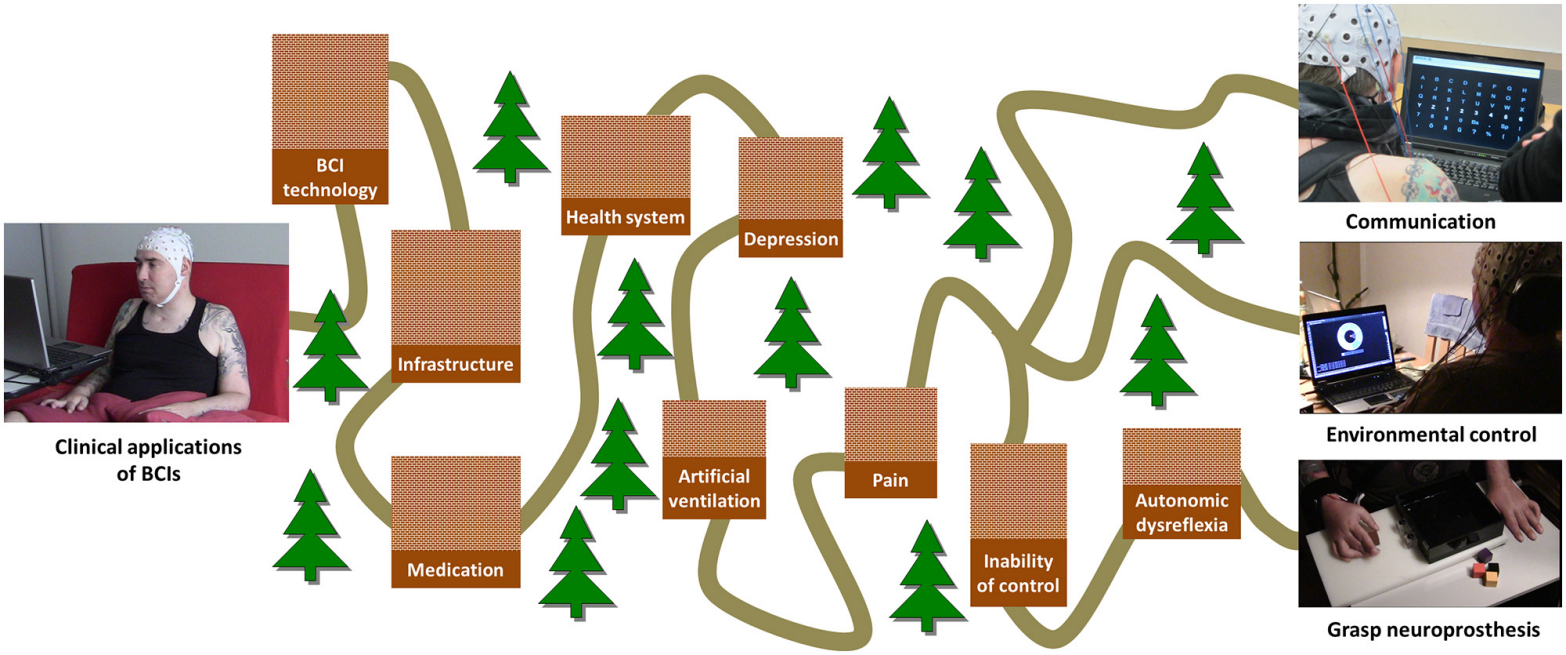
**Overview of factors limiting the successful use of different clinical BCI applications.** The “long and winding road” of clinical applications of BCI. The height of each barrier encodes its priority.

A couple of aspects have prevented BCIs so far from being regularly used as a user interface for control of assistive devices or as an adjunct therapeutic tool in the clinical setting of the rehabilitation of acute spinal cord injured patients. These limiting factors are mainly related to three distinct domains: (1) Problems and limitations of the available technology for signal acquisition and processing, (2) user-specific factors such as medication or personal user characteristics, and (3) infrastructure and health-care related constraints (**Figure 1**).

### HARDWARE AND TECHNOLOGY RELATED FACTORS

Today, commercial BCI systems are mainly based on gel electrodes placed inside an EEG cap. The correct montage of the cap and the electrode on the skull under the premise of a proper electrode contact are very time-consuming procedures taking in the case of eight electrodes an experienced therapist up to 15–20 min. With the use of more expensive active electrodes, which integrate the amplifier in the electrode, the montage time can be substantially reduced. However, if electrode gel is used, the hair of the end user needs to be washed afterward. This puts additional burden on the caregivers and the patient. Therefore, a substantial effort needs to be taken to improve the practical applicability of BCIs in clinical routine. This is related in particular to the availability of dry electrodes, which can be quickly mounted and adapted to the individual needs of a patient. Although the first technical implementations of dry or at least “one drop,” gel-less electrodes were introduced recently, it needs to be shown that they achieve the same level of signal acquisition quality in particular in an electrically noisy environment and that they do not cause any discomfort to the user (Grozea et al., 2011; Zander et al., 2011; Guger et al., 2012b).

For most effective use of time and personal resources, the necessary action of the therapist should be limited to turning the system on and off. Efforts toward this goal have recently started by implementation of a “push-button” user interface without the need for technical experts to setup and calibrate the BCI system manually (Kaufmann et al., 2012). Further improvements in terms of a higher reliability can be expected from machine learning research in BCIs, as e.g., the transfer of classifiers between individuals bears the chance to circumvent the time-consuming calibration recordings for novel users (Fazli et al., 2009), and novel algorithmic counter-measures have recently been published to adaptively cope with the non-stationarity omnipresent in brain signals (Sannelli et al., 2011; Kindermans et al., 2012; Samek et al., 2012).

### MEDICAL AND PERSONAL USER-RELATED FACTORS

#### Personal factors

During the last decade in industrial countries the mean age at the onset of SCI increased significantly from 28.7 years between 1973–1979 to 42.6 years in 2010–2012 with an ongoing trend toward more patients above the age of 65 (National Spinal Cord Injury Statistical Center, 2012). There is some evidence that the spatio-temporal brain activation patterns alter during aging and that the aging process appears to more substantively alter thalamocortical interactions leading to an increase in cortical inefficiency (Roland et al., 2011). Although, no studies exist that quantify the impact of these cortical changes on the BCI performance, it can be assumed that general cognitive problems of the older population such as attention and concentration deficits might negatively influence the ability to control or to learn how to operate a BCI.

#### Respiratory problems in high SCI

Particular in patients with high cervical lesions above C4 respiratory problems are present due to the dysfunctions of the voluntary innervation of the diaphragm and/or a thorax trauma. In the acute setting 6.5% of all patients are respirators dependent at least for some hours a day (National Spinal Cord Injury Statistical Center, 2012). 3.5% of the total population have permanent dysfunction of the respiratory function and need artificial ventilation (National Spinal Cord Injury Statistical Center, 2012). These patients are in a real need for a BCI, since other control options might not work satisfactorily. However, electrical artifacts generated by the artificial ventilator or muscular artifacts caused by shoulder elevation for voluntary ventilation support substantially decrease the quality of the EEG signals and might make a successful use of a BCI impossible.

#### Spasmolytic medication

After the period of a spinal shock spasticity evolves in the muscles in the areas of the body below the level of lesion. This inhibition of reflexes is not only apparent in skeletal muscles, but also in the detrusor muscle of the bladder resulting in episodes of incontinence. The standard medications for treatment of an overactive bladder in the first months after the SCI are anticholinergics that inhibit the receptors for acetylcholine and thereby reducing detrusor muscle tone. It has been shown that anticholinergic effects in the central nervous system can have negative influence on vigilance and concentration. While the intake of Oxybutynin leads to significant lower spectral power in all relevant frequency bands in the EEG, this effect can be avoided with Tolterodin, Trospiumchlorid, or Darifenacin (Pietzko et al., 1994; Todorova et al., 2001; Kay and Ebinger, 2008). Therefore, a careful selection of the anticholinergic medication for treatment of detrusor muscle overactivity is mandatory to prevent a detrimental effect on the performance of a BCI.

Beside anticholinergics also medication for treatment of spasticity of skeletal muscles such as baclofen, an agonist to GABA-β receptors, have an influence on the EEG spectral power distribution leading to an increase of slow brain waves (Seyfert and Straschill, 1982; Badr et al., 1983). Although systematic examinations on the influence of GABA agonists on the performance of BCI are missing, it can be assumed that the increase of slow waves and decrease of spectral components with higher frequencies will have a negative impact at least on SMR-based BCIs.

In the acute phase patients receive a high dose of medication for suppression of post-operative or trauma related nociceptive pain. A common adverse effect of this medication is its detrimental influence on attention, memory and concentration contributing to tiredness of end users. These effects alter significantly the performance of a BCI (Schreuder et al., 2013).

#### Autonomic dysreflexia

Autonomic dysreflexia is a potentially dangerous clinical syndrome that develops in individuals with SCI, resulting in acute, uncontrolled hypertension. Briefly, AD develops within the first 6 months after injury in individuals with a neurologic level at or above the sixth thoracic level (T6). AD prevalence rates vary, but the generally accepted rate is 48–90% of all individuals with injuries at T6 and above. Patients with a sensorimotor complete injury have a much higher incidence of AD (91% with complete injury vs. 27% with incomplete injury; Curt et al., 1997). The occurrence of AD increases as the patient evolves out of spinal shock. With the return of sacral reflexes, the possibility of AD increases (Schottler et al., 2009).

Autonomic dysreflexia is caused by the damage of sympathetic spinal fibers and the resulting imbalanced innervation of the autonomous nervous system, which may – if not recognized and treated correctly – lead to long-term complications such as seizures, retinal complications, pulmonary edema, myocardial infarction, or cerebral hemorrhage.

Episodes of AD can be triggered by any painful, irritating, or even strong stimulus below the level of the injury many (Krassioukov et al., 2009). Mainly bladder distension or irritations due to a blocked or kinked catheter or failure of a timely intermittent catheterization program are responsible for 75–85% of the cases (Lindan et al., 1980). AD may also be triggered by electrical stimulation of the lower extremity (Ashley et al., 1993), but has also been seen by the author in very high lesioned patients during the application of a grasp neuroprosthesis.

Although a BCI does not trigger AD, its operation may be negatively influenced by episodes of AD. Additionally, AD may prevent the successful use of a BCI-controlled neuroprosthesis either for therapeutic as well as for compensatory purposes.

#### Acute stress reaction and episodes of depression after SCI

It is a well-known fact that motivational and emotional states have an influence on the BCI performance of individuals with and without motor impairments independent of the type of BCI used (SMR or P300; Kleih et al., 2010; Nijboer et al., 2010; Hammer et al., 2012). Although, there is nothing predictable about the psychological sequelae after SCI and the response is highly individual and is mediated by both pre-morbid individual characteristics and external factors, several psychological effects occur that might heavily interfere with the successful application of a BCI (North, 1999).

The event of an SCI often occurs within minutes after a trauma or may evolve in non-traumatic causes like ischemia or infections over a few days. The affected persons are not able to slowly adapt to this novel situation, which normally results in an acute stress reaction. Generally speaking, an acute stress reaction is a transient condition that develops in response to a traumatic event. Symptoms occur within 1 month of the extreme stressor and resolves within a 4 week period. They may include a varying mixture of reduced levels of consciousness, withdrawal, anxiety symptoms, narrowing of attention, and disorientation. If the acute stress reaction persists longer than 4 weeks, an adjustment disorder may be present. Adjustment disorders may complicate the course of rehabilitation either by the decrease of compliance with the recommended medical regime resulting in an increased length of hospital stay. Common symptoms of an adjustment disorder include depressed mood, anxiety or worry, feeling unable to cope with life at present or plan ahead, stress-related physical symptoms such as headaches and interference with social functioning or performance of daily activities.

Although, results from systematic investigations on this issue are missing, an acute stress reaction negatively impacts the use of BCIs in patients during the very acute phase up to 4 weeks after the injury.

Additionally to the psychological complication mentioned so far, patients may experience episodes of depression already a few weeks after the injury. Depression is more common in the SCI population compared the general population. Estimated rates of depression among people with SCI range from 11 to 37% (Craig et al., 2014). Common emotional, behavioral, and physical symptoms of major depression are markedly depressed mood, loss of interest, reduced self-esteem and self- confidence, feelings of guilt and worthlessness, reduced energy leading to fatigue, diminished activity, and reduced concentration. All of those symptoms may result in an unwillingness to participate in any kind of rehabilitative training including BCI therapy. Patients suffering from a major depression refuse to be provided with assistive technology in general.

There is also evidence that the P300 amplitude is decreased in individuals with major depression (Diner et al., 1985), which might contribute to the inability to achieve a sufficient level of BCI performance. The inability of BCI control might in turn contribute to an increase in the symptoms of depression. To avoid this vicious circle a thorough neuropsychological assessment is needed in acute patients to identify any signs of major depression.

#### SMR-based BCIs and neuropathic pain

Pain is a major problem after SCI and most of the patients report to have pain. In the acute phase after an SCI it is mainly nociceptive pain due to trauma or spams (Finnerup, 2013). Usually within the first year after the injury neuropathic pain develops in about 40–50% of the patients and tends to become chronic (Siddall et al., 2003). Beside the general negative effects of pain on the quality of life of the affected persons, pain leads to deficits in concentration and attention – both having negative impact on the BCI performance. A recent study showed that the EEG activity of spinal cord injured patients with chronic neuropathic pain differs to that of spinal cord injured patients with no pain and also to that of able-bodied people (Vuckovic et al., 2014). Frequency-specific EEG signatures were identified that may be used to monitor the development of neuropathic pain. However, it is not clear if the evolvement of these EEG patterns have a detrimental effect on BCI control.

For operation of an SMR-based BCI users have to imagine movements from different, also paralyzed parts of the body. The influence of MI on neuropathic pain is still an issue of debate and it is not entirely clear, if MI training is lowering or increasing the perceived pain level. It was shown in patients with a chronic thoracic SCI that imagination of foot movements three times a day for a period of 7 days increases neuropathic pain (Gustin et al., 2008). In contrast to this, preliminary studies suggest that neurofeedback has the potential to help patients with otherwise refractory chronic pain (Jensen et al., 2013a). Recent findings indicate that certain EEG activity patterns may be associated with more pain or a vulnerability to experience chronic pain in persons with SCI. Research examining the extent to which changes in this EEG activity may result in pain relief is warranted (Jensen et al., 2013b).

In summary, the use of neurofeedback for prevention of chronic neuropathic pain is still controversial. Clinical studies are urgently needed to reveal if BCIs represent a promising tool to prevent the development of neuropathic pain in SCI.

#### Inability for BCI control

While BCIs based on the registration of P300 (Guger et al., 2009a) and SSVEPs (Guger et al., 2012a) can be operated by a vast majority of users, it is well-known that SMR-BCIs are not suitable for all users. In up to one third of the non-motor-impaired participants the BCI is unable to detect classifiable task related EEG patterns (Guger et al., 2003). Consequently, these subjects cannot quickly be provided with a BCI-controlled application or need at least a substantial amount of training for sufficient operation of a BCI. The causes for this inability for controlling a BCI (other synonyms are BCI-“inefficiency,” BCI-aptitude) have not yet been satisfactorily described. The few studies that explicitly investigated the predictive value of user- and BCI-related factors on BCI performance have been performed with subjects without motor impairments (Kübler et al., 2004; Blankertz et al., 2010; Halder et al., 2011; Holz et al., 2011; Kaufmann et al., 2013). Thus, it is not known, in how far these results are representative also for people with motor impairments such as spinal cord injuries.

In a recent study, a three-class MI screening (left hand, right hand, feet) was performed with a group of 10 able-bodied and 16 tetra- and paraplegic people with a complete SCI with the objective of determining what differences were present between the user groups and how they would impact upon the ability of these user groups to interact with a BCI. Although, the patient group was very heterogeneous in terms of time after trauma and age it is seen that both the tetraplegic and paraplegic patients have some significant differences in event-related desynchronization strengths, exhibit significant increases in synchronization and reach significantly lower mean accuracies (66.1%) than the group of non-impaired subjects (85.1%; Müller-Putz et al., 2014).

In another study, authors compared the BCI performance of 15 end users with complete SCI, eight of them paraplegic and seven tetraplegic (Pfurtscheller et al., 2009). It was found that five of the paraplegic individuals had a mean accuracy above 70% but only one tetraplegic person achieved this performance level. The reason for this observation is still unclear. It can be speculated that the missing sensory loop restricts the vividness of the imagined movements and therefore the performance. This statement is supported by (Alkadhi et al., 2005), who showed the positive correlation of cortical activation and vividness of the imagined movement.

It is a well-accepted statement in the BCI community, that training is expected to improve the performance of SMR-BCIs. Data on the course and performance of long-term MI-BCI training in individuals with chronic high-level SCI is sparse. In one study, two C4, three C6 and four C7 end users were trained to operate an MI-BCI with the goal of controlling a robotic arm (Onose et al., 2012). The average performance of all subjects was quite moderate, determined as 70.5%. In three of the subjects the online performance was up to 20% worse (in a two-class task) than the oﬄine performance. Unfortunately, the authors did not explicitly state how many oﬄine runs were used for classifier training, so it is possible that their classifiers were trained too intensively on the same dataset. This may result in overfitting and therefore suggesting a far higher oﬄine performance than actually achieved during online trials. Furthermore, online experiments are more demanding, which may also affect the performance. One of the study subjects fell asleep during the training, which indicates a high physical and mental workload during the operation of the BCI.

In the framework of a single case study, in which an individual with a lesion of the upper cervical spinal cord was provided with a BCI-controlled upper extremity neuroprosthesis, no training effects occurred over a training time of more than 6 months. Even after 415 MI-BCI runs, the end user’s average performance did not show any trend toward improvement, but remained at about 70% with large day-to-day variances. This moderate average performance may be explained by the significant differences in movement-related ß-band modulations found in subjects with SCI as compared to non-injured individuals (Gourab and Schmit, 2010). In detail, a correlation seems to exist between decreased ERS amplitude and the severity of the impairment of the limb in which the movement was attempted. This supports the view that in high-level tetraplegic subjects, an extensive BCI training period does not necessarily lead to superior results. Although, this statement has to be validated in future studies with a larger population, it must be clearly communicated to patients with an acute SCI. It is entirely possible that only low to moderate performance will be achieved with the danger of causing additional sadness or depression and generating a higher stress level, because severely motor impaired persons may get the impression that in addition to their body even their brains do not work properly.

### INFRASTRUCTURE AND HEALTH-CARE SYSTEM RELATED FACTORS

Beside BCI and user-related factors there are factors associated to the typical infrastructure in clinics and to the health-care system in general, which form major barriers for the successful integration of BCIs into clinical routine. Patients rehabilitated in industrial countries take part in normally two sessions of physio- and one session of occupational therapy of a length of 30 min each. With the currently available BCI technology a BCI session takes at least 1 hour to setup the BCI, perform a supervised training/operation and remove the gel from the hair of the patients. Additionally, a BCI needs to be set up and adapted to each individual user, which takes even more time in particular during the first sessions. This means that patients will at least miss two out of three daily sessions of conventional therapy, which is neither accepted by the clinical staff nor by the patients themselves. Therefore, BCIs are likely to be used as adjunct rehabilitative tools with the need for additional personnel or therapy slots. However, these BCI application sessions are not separately reimbursed by the health service or insurances and need to be covered by the budget of the clinics themselves.

The major problem in the field of BCIs is that randomized controlled trials providing clear evidence for their superiority compared to traditional approaches are missing completely (Kübler et al., 2013). In particular, the relationship between the investments in terms of personnel, time and money and the degree of improvement in patient outcomes needs to be determined. This information is mandatory to initiate a dialog with health service payers with the aim of reimbursement of BCI applications during the inpatient rehabilitation phase and later on in the chronic stage also at home.

At this point it must be emphasized that general recommendations on the integration of novel therapies such as the BCI into clinical routine cannot be made due to huge differences in the length of primary rehabilitation between health systems of different countries and in the modes of reimbursement in particular in different European countries.

## CONCLUSION AND OUTLOOK

In the context of rehabilitation of individuals with SCI in the acute and subacute stage non-invasive BCIs represent a valuable adjunct to traditional compensatory and restorative approaches in the clinical setting. The main focus of their application is the use as an additional or alternative channel for operation of assistive devices enabling communication and environmental control in patients with very high lesions of the spinal cord. For this application P300-based BCI systems are the first choice, because almost all persons are able to achieve a sufficient level of control with only a small amount of training. MI based BCIs providing a feedback on the modulation of SMRs of the primary motor cortex may evolve to an exciting adjunct to conventional neurorehabilitative therapies aiming at enhancement of motor function by guidance of neural plasticity. This approach is particularly promising, if combined with neuroprotheses of the upper extremity providing a strong proprioceptive feedback. However, clinical studies need to show that no detrimental effects like an increase of neuropathic pain occur during this type of training.

On a more general level, a couple of factors are limiting the successful use of BCIs, among them technology related, user specific and infrastructure dependent factors. The major limitations in the technological domain are the need for gel electrodes with their time-consuming and non-user friendly handling and the need for technical experts for setup and supervision of the BCI. Additionally, user related issues such as spasmolytic and other medication, acute stress syndromes, or episodes of depression may have a negative impact on the BCI performance with the risk of causing additional frustration and sadness. Limited personnel and time resources are a general problem for successful implementation of any kind of novel therapeutic approach in the clinical setting. These may be overcome by regular reimbursement of BCI therapies in the clinical setting. However, to achieve this large scale clinical trials need to be performed, which prove the efficacy and additional benefit of BCIs.

Studies involving individuals with isolated injuries of the spinal cord may provide preliminary information on the feasibility of BCI-based neurorehabilitative approaches in other neurological patient groups like stroke survivors or patients with traumatic brain injury. The challenges and general problems seen in studies with individuals with SCI in the clinical environment are likely to occur also in other patient groups and help to realistically estimate the number of potential end user of BCI technology.

## Conflict of Interest Statement

The author declares that the research was conducted in the absence of any commercial or financial relationships that could be construed as a potential conflict of interest.
